# The Impact of the Introduction of MELD on the Dynamics of the Liver Transplantation Waiting List in São Paulo, Brazil

**DOI:** 10.1155/2014/219789

**Published:** 2014-11-27

**Authors:** Eleazar Chaib, Eduardo Massad, Bruno Butturi Varone, Andre Leopoldino Bordini, Flavio Henrique Ferreira Galvão, Alessandra Crescenzi, Arnaldo Bernal Filho, Luiz Augusto Carneiro D'Albuquerque

**Affiliations:** ^1^Division of Liver Transplantation, LIM 37, Department of Gastroenterology, São Paulo School of Medicine, Suite 3206, 3rd Foor, 01246-903 São Paulo, SP, Brazil; ^2^Department of Informatics, LIM 01, University of São Paulo School of Medicine, 01246-903 São Paulo, SP, Brazil

## Abstract

Until July 15, 2006, the time on the waiting list was the main criterion for allocating deceased donor livers in the state of São Paulo, Brazil. After this date, MELD has been the basis for the allocation of deceased donor livers for adult transplantation. Our aim was to compare the waitlist dynamics before MELD (1997–2005) and after MELD (2006–2012) in our state. A retrospective study was conducted including the data from all the liver transplant candidate waiting lists from July 1997 to December 2012. The data were related to the actual number of liver transplantations (*Tr*), the incidence of new patients on the list (*I*), and the number of patients who died while being on the waitlist (*D*) from 1997 to 2005 (the pre-MELD era) and from 2006 to 2012 (the post-MELD era). The number of transplantations from 1997 to 2005 and from 2006 to 2012 increased nonlinearly, with a clear trend to levelling to equilibrium at approximately 350 and 500 cases per year, respectively. The implementation of the MELD score resulted in a shorter waiting time until liver transplantation. Additionally, there was a significant effect on the waitlist dynamics in the first 4 years; however, the curves diverge from there, implying a null long-range effect on the waitlist by the MELD scores.

## 1. Introduction

The global liver allocation system in use until 2002 was based on the Child-Turcotte-Pugh (CTP) scale and other scores as well as on the waiting time; the system became the major discriminator of patients on the waitlist without reflecting their actual liver dysfunction. The system for prioritising adult patients on the waitlist in the USA has since changed from a status-based algorithm using the CTP scale to a system using a continuous model for end-stage liver disease (MELD) [1]. The MELD score was originally developed to predict the survival after a transjugular intrahepatic portosystemic shunt procedure (TIPS) [2].

In São Paulo, the time on the waiting list was the primary criterion adopted to allocate deceased donor livers until July 15, 2006. After this date, MELD was the basis for the allocation of deceased donor livers for adult transplantation. The MELD score primarily sought to increase access to transplantation for severely ill patients as a means to reduce the mortality rate of the waiting list patients. The MELD score does not consider the posttransplant benefit.

Our aim was to compare the waitlist dynamics in the pre-MELD (1997–2005) and post-MELD (2006–2012) periods in the state of São Paulo, Brazil.

## 2. Material and Methods

A retrospective study was initially conducted that included the waiting list data of all the liver transplant candidates from July 1997 to December 2012 in the state of São Paulo, Brazil. The data were from the liver transplant research database of the Health Secretariat of São Paulo.

In this study, inclusion was restricted to adult patients (>18 years) who were candidates for liver transplantation. Living-donors related liver transplantation cases and split-livers and paediatric recipients were excluded from the study. The candidates were divided into the pre-MELD group for those listed from July 2007 to December 2005 and the post-MELD group for those listed from July 2006 to December 2012. The patients with hepatocellular carcinoma (HCC) according to the Milan criteria were granted additional MELD points. On the first year after the implementation of MELD, these patients arbitrarily received a MELD score of 29, which was changed to a MELD score of 24 in the second year after the implementation of MELD.

The patients who had acute hepatic failure and were not allocated by the MELD system were excluded. The Organ Procurement Organization (OPO) obtains the waiting list additions, modifications, and removals directly from the transplant centres. Patients were removed from the waitlist for the following major reasons: clinical improvement without a transplant; death; or being unable to receive a transplant based on the health status of the patient.

MELD was calculated at the time of transplantation, as previously described [3], using the following objective variables: the serum creatinine, bilirubin, and INR levels. The minimum acceptable value for INR, creatinine, and bilirubin is 1. The maximum acceptable value for serum creatinine is 4 mg/dL. If the patient had been dialysed twice within 7 days, then the value for the serum creatinine would be 4. The maximum value for the MELD score is 40. The laboratory data and the MELD score were not collected in the pre-MELD era. For those who received a transplant after the implementation of the MELD system, the MELD score at transplantation was used as a marker of severe liver disease.

The data related to the actual number of liver transplantations (*Tr*), the incidence of new patients on the list (*I*), and the number of patients who died while on the waitlist (*D*) from 1997 to 2005 (the pre-MELD era) and from 2006 to 2012 (the post-MELD era) are shown in Tables 1 and 2, respectively.

We used the (*Tr*) data from Table 1 (1997–2005) and Table 2 (2006–2012) fitting a continuous curve by the maximum likelihood method [4] to project the number of future transplantations (*Tr*). The pre-MELD era waitlist dynamics were previously described by our group [5]. The resultant equations are as follows:

(1) $$\begin{array}{c} Tr=107.07\;\left(\text{year}\right)+72.943,\;\;Tr=500\;\left(\text{year}\right)+184.7, \end{array}$$

for the first (1997–2005) and second (2006–2012) groups, respectively.

## 3. Results

**Figure 1:**
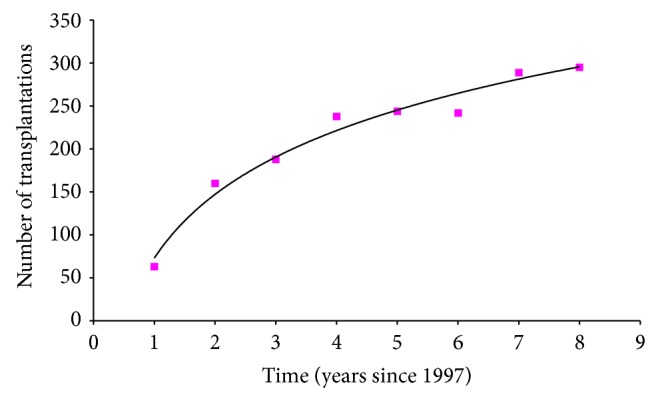
Fitted curve by the method of maximum likelihood to the data from Table 1 to project the number of transplantations, *Tr*, in the future (before MELD).

**Figure 2:**
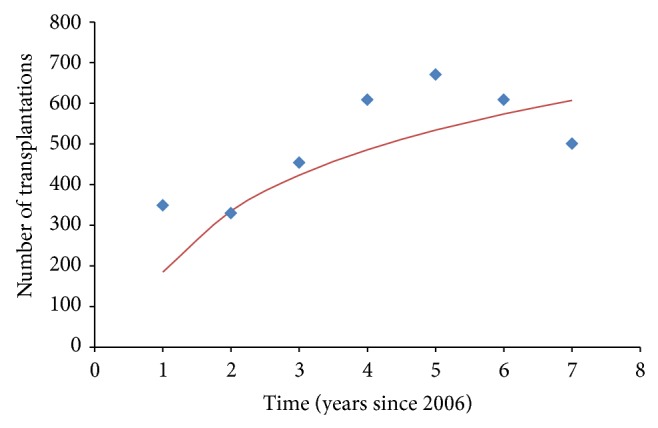
Fitting curve by the method of maximum likelihood [4] to the data from Table 2 to project the number of transplantations, *Tr*, in the future (after MELD).

The results are visualised in Figures 1 and 2, in which the number of transplants performed is fitted to the above function used to project the list size. The number of transplants from 1997 to 2005 and from 2006 to 2012 increased nonlinearly, with a clear trend to levelling to an equilibrium of approximately 350 and 500 cases per year, respectively.

We projected the size of the waiting list, *L*, by considering the incidence of new patients per year, *I*; the number of transplants performed in that year, *Tr*; and the number of patients who died while being on the waiting list, *D*. The dynamics of the waiting list is presented by the following difference equation:

(2) $$\begin{array}{c} L_{t+1}=L_{t}+I_{t}-D_{t}-Tr_{t} \end{array}$$

with the list size at time *t* + 1 being equal to the size of the list at time *t*, plus the new patients added to the list at time *t* minus those patients who died while on the waiting list at time *t* and minus those patients who received a graft at time *t*. The variables, *I* and *D*, from 2006 were projected by fitting an equation by the maximum likelihood, in the identical manner as the equation was fitted for *Tr*.

**Figure 3:**
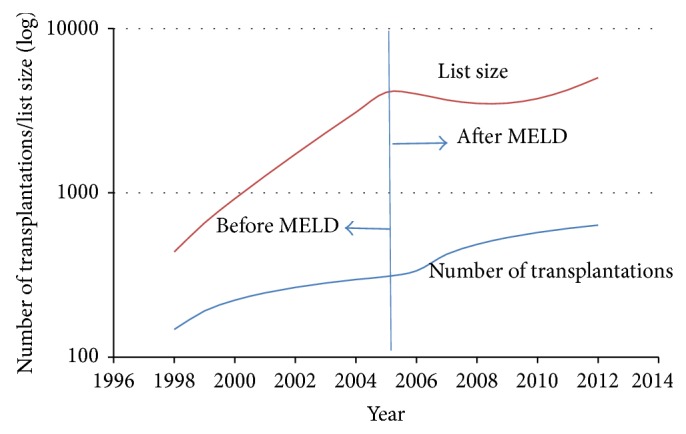
Fitting of (2) to the data from Tables 1 (before MELD) and 2 (after MELD).

The introduction of the MELD score had a significant effect on the waitlist dynamics in the first 4 years after its introduction; however, the curves diverge from there, implying a null long-range effect by the MELD scores on the waitlist (Figure 3).

**Figure 4:**
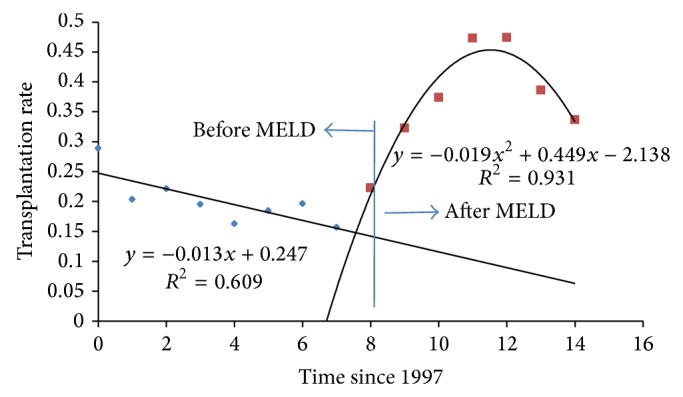
Transplantation rate (proportion of those patients transplanted with respect to those who enter the waiting list per time unit), comparing the pre- and post-MELD periods.

Additionally, we calculated the net transplantation rate, defined as the number of patients who received a graft among all the patients on the waiting list per time unit before and after the introduction of the MELD score. This calculation was performed by dividing the variables *Tr* by *I* from Table 2. The result is shown in Figure 4. The transplantation rate decreased until 2005, increased dramatically thereafter, and peaked at approximately 50% in 2009/2010, dropping from there onwards.

## 4. Discussion

The increased mortality of patients waiting for a liver transplant and the shortage of donor organs induced efforts to improve the allocation criteria for liver transplantation candidates. The introduction of the MELD system in the USA for graft allocation resulted in a 3.5% reduction in waitlist mortality whereas the early-stage survival of liver transplant recipients remained unchanged, despite the selection of more seriously ill patients for transplantation [6].

Although MELD eliminates subjective assessments and shows accuracy for predicting the outcome in patients with decompensated cirrhosis, it has several limitations [7, 8]. One of the limitations of the MELD score is that the components of the MELD score were found to independently and individually predict death on the waitlist.

The major reason for MELD implementation was to decrease the number of deaths of waitlist patients, providing each patient an identical probability of receiving a transplant at presumed fixed condition levels. Previously, priority was determined by a more complex system, in which the waiting list time and patient condition, classified in a semiquantitative way, were linked (the presence of encephalopathy and ascites as well as the waiting time and patient location). An ultimate goal has been to end the privilege of selecting the candidate on a clinical basis (considering various parameters such as the primary disease, degree of residual liver function, extrahepatic involvement, waiting list time, and donor related risk), which was once a prerogative of the transplant surgeon.

The role of the match between the “donor quality” and the severity of the recipient's disease has not been completely investigated. In many transplant centres, standard livers are routinely transplanted in low-risk patients, whereas marginal donors are reserved for high-risk patients. Our group suggested that better results are obtained if the risk related to the donor and the risk related to the recipient are not merged.

The effectiveness of MELD as a prognostic index has been fully validated in cirrhotic patients waiting for transplantation. The role of MELD as a prognostic index in liver transplant patients is controversial. The efficacy of MELD in predicting graft survival has been reported in several uncontrolled single centre cohorts characterised by an intrinsic high-risk condition [9].

A major challenge facing the field of liver transplantation is the critical shortage of donor organs, which has led to a dramatic increase in the number of patients on the waitlist as well as in the waiting time of the patients. In the pre-MELD era, the number of liver transplantations increased 1.86-fold (from 160 to 299) from 1998 to 2005; however, the number of patients on the liver waitlist increased 3.44-fold (from 553 to 1907). The number of deaths of the waitlist patients increased 2.06-fold (from 321 to 662, Figure 1).

The implementation of the new liver allocation system in our state has required a change in the disease severity score, with minimal weighting being allocated to the waiting time compared with the previous system that was based on the Child-Turcotte-Pugh score and on the waiting time.

The lower priority placed on the waiting time has improved organ access for worse transplant candidates, and worse patients were selected from the pretransplant waiting list. This fact is reflected by the significant increase of the median MELD score at the time of liver transplantation as well as by the decreased median waiting time. We found that the median time on the waitlist decreased only for the patients in whom LT was performed whereas a significant proportion of patients with lower MELD scores are likely to have much longer waiting times.

After the implementation of MELD (2006), we observed that the number of liver transplants increased 1.43-fold (from 349 to 501) from 2006 to 2012; the number of patients on the liver transplantation waitlist was slightly reduced (0.95-fold), from 1566 to 1488 patients. The number of deaths of waitlisted patients has been significantly reduced (2.02-fold), from 895 to 441 patients (Figure 2).

The major controversy following the implementation of MELD is the balance in organ allocation between reduced waitlist mortality and the best posttransplantation outcome [9]. Numerous studies have investigated, with varying results, the prognostic value of the MELD score for early and late posttransplant survival [3, 10, 11]. At our centre, the recipients with a MELD score between 20 and 29 received organs fulfilling at least one extended donor criterion significantly more frequently. After the implementation of MELD, rating patients with a higher score based on longer waiting times became meaningless, and acceptance of an organ from extended criteria donors via centre-based allocation represents the only opportunity for transplantation.

In conclusion, the implementation of the MELD score resulted in a shorter waiting time until liver transplantation for patients. The MELD system had a significant effect on the waitlist dynamics in the first 4 years; however, the curves diverge from that point, implying a null long-term effect by the MELD scores on the list of patients waiting for transplantation.

## Figures and Tables

**Table 1:** Actual number of liver transplantations (1997–2005) (*Tr*), the incidence of new patients on the list (*I*), and the number of patients who died while being on the waiting list (*D*), in the state of São Paulo from 1997 until 2005 (before MELD).

Year	*Tr*	*I*	*D*
1997	63	—	—
1998	160	553	321
1999	188	923	414
2000	238	1074	548
2001	244	1248	604
2002	242	1486	725
2003	289	1564	723
2004	295	1500	671
2005	299	1907	662

**Table 2:** Actual number of liver transplantations (2006–2012) (*Tr*), the incidence of new patients on the list (*I*), and the number of patients who died while being on the waiting list (*D*), in the state of São Paulo from 2006 until 2012 (after MELD).

Year	*Tr*	*I*	*D*
2006	349	1566	895
2007	330	1022	734
2008	454	1213	490
2009	609	1287	455
2010	671	1415	403
2011	609	1577	470
2012	501	1488	441
